# Regenerative Endodontic Treatment of a Maxillary Mature Premolar

**DOI:** 10.1155/2018/5234136

**Published:** 2018-01-18

**Authors:** Qingan Xu, Zhou Li

**Affiliations:** ^1^School of Nursing and Medical Technology, Jianghan University, Wuhan, China; ^2^Wuhan First Stomatology Hospital, Wuhan, China; ^3^Department of Pediatric Dentistry, Stomatology Hospital of Guangzhou Medical University, Guanghzou, China

## Abstract

Regenerative endodontic treatment was performed on a mature maxillary premolar diagnosed as chronic pulpitis. The root canals were chemomechanically prepared and placed intracanal medicaments at the first appointment. Then 2 weeks later, a blood clot was created in the canals, over which mineral trioxide aggregate was placed. At 6-month follow-up, cementum-like tissue seemed to be formed in the root canal along with nearly recovered pulp vitality. At 12-month recall, the radiographic results revealed evidence of root wall thickening. At 30-month recall, no periapical lesion was found. This case report indicates that regenerative endodontic treatment for the mature premolar is feasible. More cases are needed for further validation.

## 1. Introduction

Regenerative endodontics is the use of biologically based procedures designed to replace damaged structures, including dentin and root structures, as well as cells of the pulp-dentin complex [1]. It shows a broad prospect in both tissue engineering and clinical application, which was adopted as a procedure code by the American Dental Association in January 2011 [2].

Traditionally, for pulpitis or apical periodontitis, root canal treatment (RCT) is the most common treatment method. However, RCT results in complete removal of pulp tissues, which means loss of a warning system, immunoresponse and formation of dentin as active defense mechanisms against invading toxins and bacteria, and cease of root development in the cases of young patients. Moreover, in some special cases, the absence of an apical constriction makes RCT problematic. Apexification also has some disadvantages such as variability of treatment time, patient compliance, and increased risk of tooth fracture, which may do not promote the continued development of the root and just “closed” the open apex by a calcific barrier. In contrast, the goal of regenerative endodontics is to induce biologic replacement of dental tissues and regenerate a functional and healthy pulp-dentin complex. So, regenerative endodontic treatment may be a good alternative to conventional endodontic therapies, especially for immature permanent teeth.

To date, more and more case reports and case series showed the feasibility of regenerative endodontic treatment [3–5]; however, most of these cases were performed on immature teeth. Recently, cases on mature teeth with closed apex were occasionally reported [6, 7]. In the present report, we described the treatment of a mature tooth with pulpitis by using regenerative endodontic therapy, and the follow-up period lasted for 30 months.

## 2. Case Report

**Figure 1:**
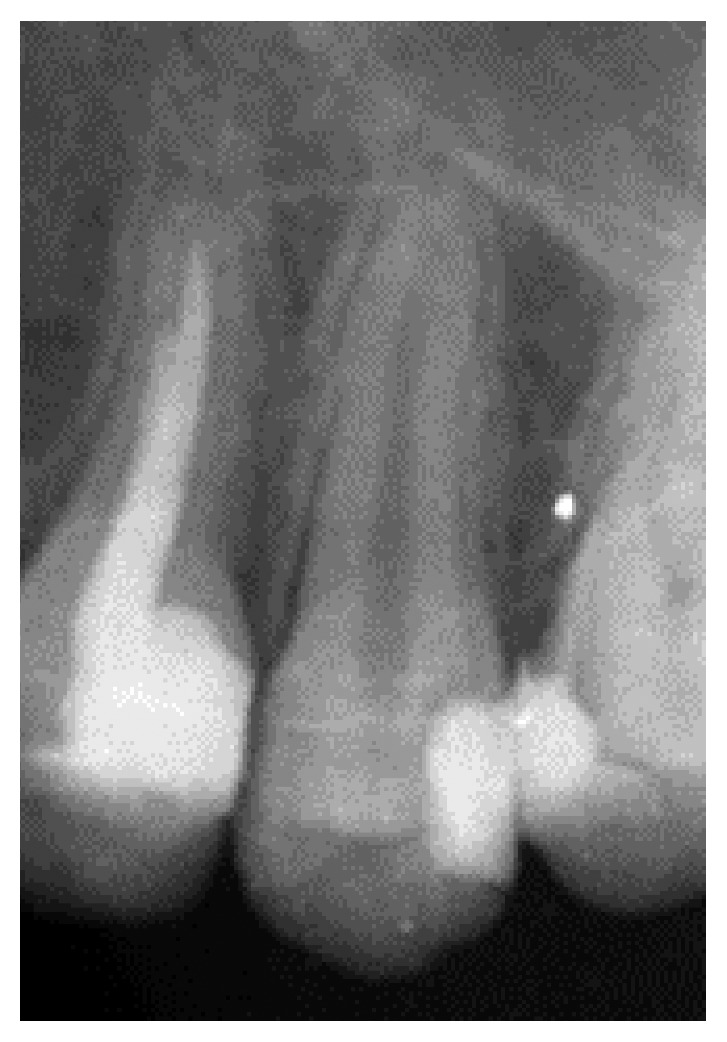
Preoperative radiograph showing a filling shadow close to the pulp and a fully developed root and closed apex of tooth #13.

A 15-year-old girl in good health presented to the Department of Endodontics, Wuhan University Hospital of Stomatology, in November, 2014, with a chief complaint of cold water pain on her left upper posterior tooth for the last 5 days. Three months earlier, she had caries filled in the same region. Intraoral examination revealed that tooth #13 had an existing distal occlusal composite restoration. Tooth #13 showed sensitivity to pulp test and no sensitivity to palpation and percussion. Radiographic examination showed distal a filling shadow close to the pulp and a fully developed root and closed apex (Figure 1). On the basis of the results of the clinical and radiographic examinations, the diagnosis for tooth #13 was chronic pulpitis.

An informed consent was obtained from the patient and her parent for regenerative endodontic treatment. An access cavity preparation was performed after local anesthesia with 2% lidocaine and rubber dam isolation. The canal was irrigated copiously with 5.25% sodium hypochlorite and 17% EDTA. The canal was partially dried with sterile paper points and then dusted with a mixture powder of ciprofloxacin and metronidazole. The tooth was then temporized with zinc oxide eugenol cement.

**Figure 2:**
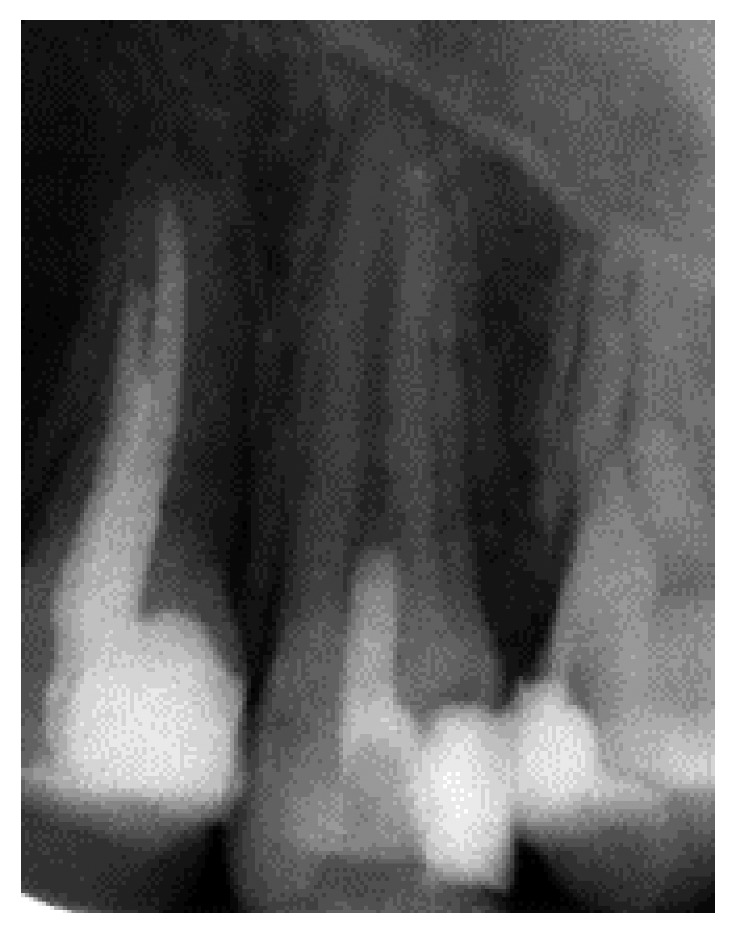
Postoperative radiograph presenting placement of the MTA and glass ionomer cement.

At 2-week follow-up, the patient was asymptomatic. The tooth was not sensitive to percussion and palpation. After local anesthesia by using 2% lidocaine and rubber dam isolation, the temporary restoration was removed. The antibiotic mixture was completely removed with 5.25% sodium hypochlorite. The apical foramen was enlarged up to 0.6 mm with a size #60 hand file (K-file). Seventeen percent EDTA was used as a final irrigating agent and left in the canal for about 1 minute. Then, the canal was dried with sterile paper points. A size #25 K-file was introduced into the root canal to irritate the periapical tissue and to create some bleeding into the root canal. The mineral trioxide aggregate (MTA) was placed approximately 2 mm below the cementoenamel junction. The tooth was restored with glass ionomer cement (Figure 2).

**Figure 3:**
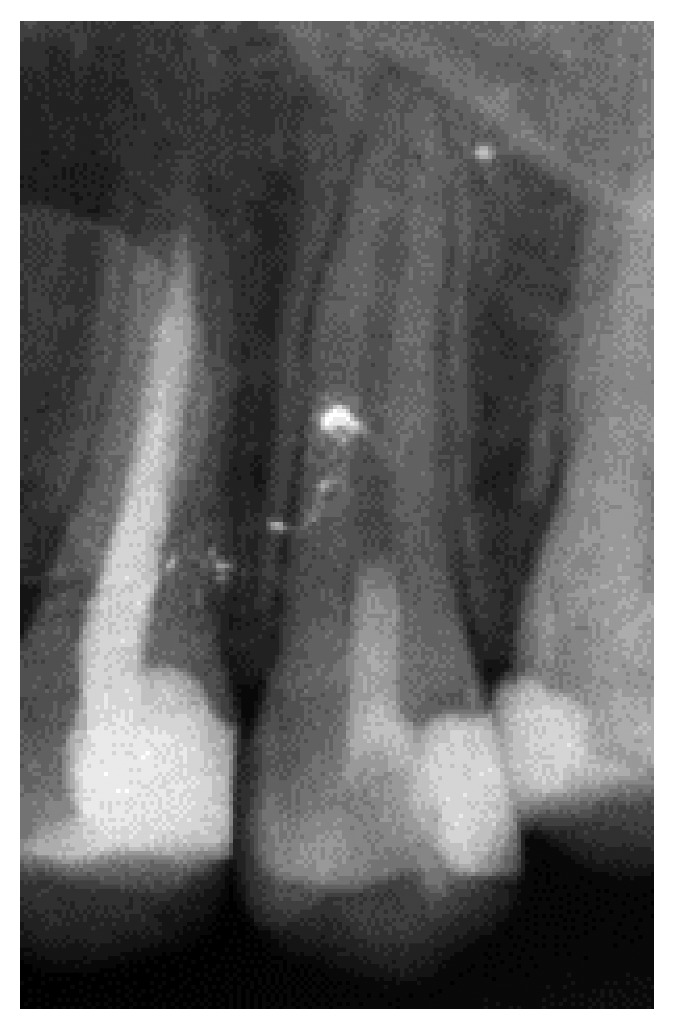
Three-month follow-up radiograph showing a normal periodontal ligament space.

At 3-month recall, the patient was asymptomatic. The tooth was not sensitive to percussion or palpation and not responsive to pulp testing. The radiograph showed nearly a normal periodontal ligament space (Figure 3). The tooth was restored with composite resin after removal of the glass ionomer cement.

**Figure 4:**
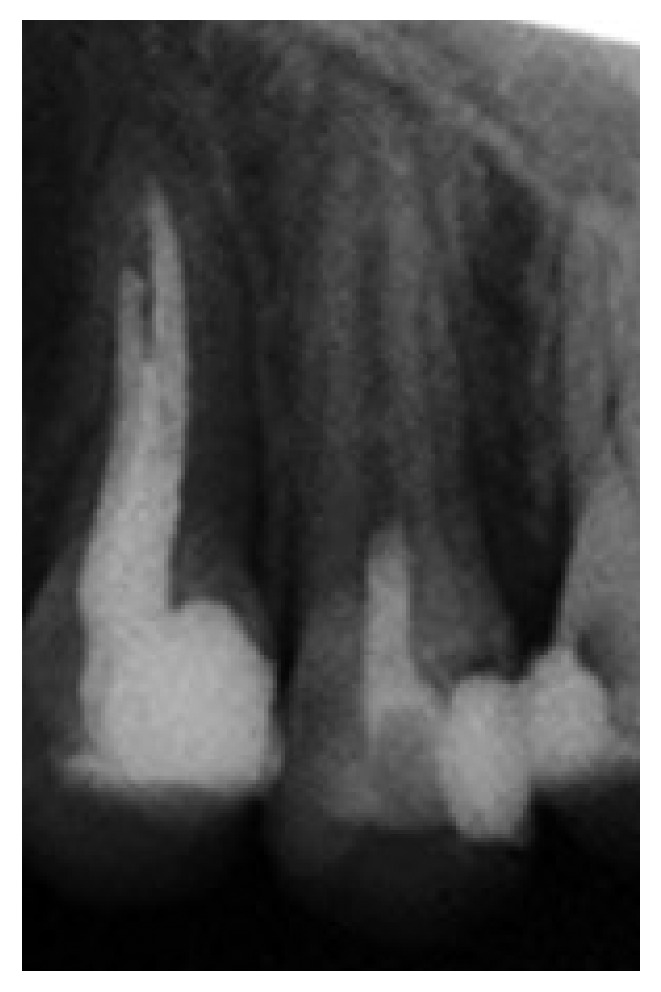
Six-month follow-up radiograph showing narrowing of the canal space with radiopaque shadows.

At 6-month follow-up, tooth #13 regained pulp sensibility and responded positively to the electric pulp tester (EPT) and Endo-Ice at a similar extent as the control tooth. The radiographic results showed narrowing of the canal space with radiopaque shadows (Figure 4).

**Figure 5:**
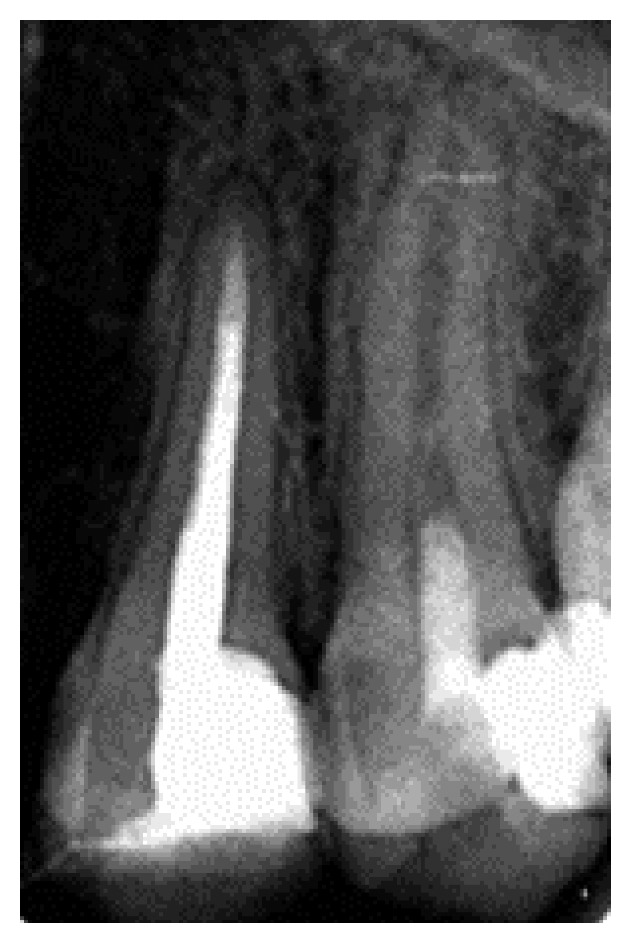
Twelve-month follow-up radiograph showing root wall thickening.

At 12-month recall, there was no pain on percussion and palpation. Tooth #13 was normally responsive to Endo-Ice and EPT. The radiographic results revealed evidence of root wall thickening (Figure 5).

**Figure 6:**
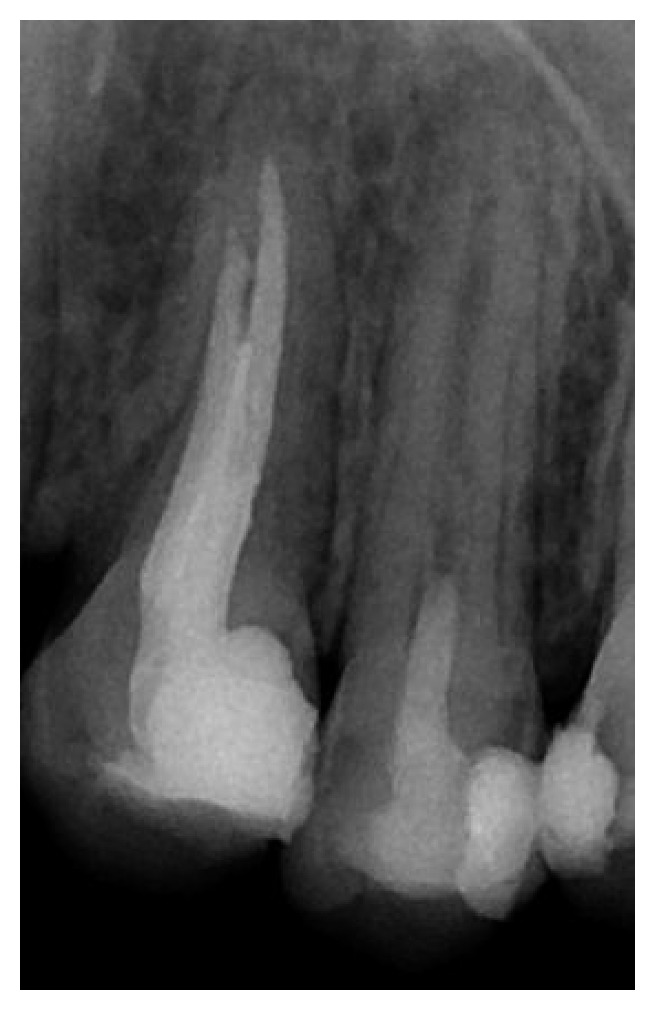
Thirty-month follow-up radiograph showing a normal periapical image.

At 30-month recall, tooth #13 still showed normal pulp vitality compared to the control tooth, and no periapical lesion was found on the X-ray film (Figure 6). The recovery of pulp vitality at different follow-up time points was summarized in Table 1.

## 3. Discussion

Age of thirteen to fifteen years is the period of a premolar maturation, during which the apex is gradually closed. In this case, the patient was 15 years old. The X-ray showed that the apex was fully developed. Furthermore, during the regenerative endodontic treatment, a #15 K-file was used to probe the apical foramen and an obvious sense of friction was found, so we judged the tooth in this case belongs to mature permanent tooth. Apical bleeding was induced into the root canal, leading to a suitable environment for pulp regeneration with an enrichment of host endogenous stem cells and growth factors in a bioactive scaffold. The new-grown tissue was not a pulp tissue revealed by the calcified shadows in the root canal. Instead, it might be a newly generated cementum-like tissue termed herein “intracanal cementum” as reported in relevant literature [8].

The concept of regenerating pulpal tissue was promulgated by the classic studies of Nygaard-Ostby in 1961. His early research revealed tissue repair (fibroblasts, collagen, and sparse vascularity) without histologic evidence of regeneration of the pulp-dentin complex, while a newly mineralized tissue appeared to be cementum was evident on some dentinal walls. The second case series 10 years later showed histologic evidence of an ingrowth of vascularized fibrous connective tissue in the most of the teeth. Wang et al. [8] analyzed the histologic characterization of regenerated tissues in the canal space after the revitalization/revascularization procedure of immature dog teeth with apical periodontitis. It is found that the canal dentinal walls were thickened by the apposition of a newly generated cementum-like tissue which was similar to cellular cementum. One of the cases showed partial survival of pulp tissue juxtaposed with fibrous connective tissue that formed intracanal cementum on canal dentin walls. Bone or bone-like tissue, the so-called intracanal bone, and connective tissue similar to periodontal ligament were also present in the canal space surrounding the intracanal cementum and/or intracanal bone. In the present case, the intracanal cementum may be able to explain the imaging manifestations well, including the radiopaque shadows and root wall thickening. But further studies are still needed to prove whether the recovery of pulp vitality means there is regeneration of nerve fibers and whether there are blood vessels, fibroblast, newly formed dentin, etc.

It is believed that stem cells play an important role in histogenesis as well as pulp regeneration. To our knowledge, at least five types of postnatal mesenchymal stem cells can differentiate into odontoblast-like cells based upon the studies of recent years, including dental pulp stem cells (DPSCs), stem cells of human exfoliated deciduous teeth (SHED), dental follicle progenitor cells (DFPCs), stem cells of the apical papilla (SCAP), and bone marrow-derived mesenchymal stem cells (BMMSCs). Promisingly, studies in vivo have shown that transplantation of stem cells can yield ectopic dental pulp-like tissues in tooth slices or fragments. However, no clinical trials of stem cell transplantation have been reported to date. It has been adopted by the American Dental Association (ADA) to induce apical bleeding into the root canal in immature permanent teeth with necrotic pulps which have been extirpated in January 2011 [9]. The evoked bleeding during endodontic regenerative procedures conducted on immature teeth reveals a massive influx of mesenchymal stem cells into the root canal space. In contrast to cell-based approaches of stem cell transplantation and/or tissue engineering, this “induced bleeding” technique was named “cell-free approach” [10], which was applied in our present case as well.

Scaffold is thought of as an indispensable element for pulp/dentin regeneration. In this report, sterile blood clot induced into the root canal also acted as the 3-dimensional scaffold [11] for tissue ingrowth which supplied potent chemoattractants and growth factors simultaneously to induce cell migration, proliferation, and differentiation. Therefore, no other scaffold materials or growth factors were used in this case while considering noninvasion and avoiding rejection. But they may be the substitution when ideal blood clot is absent. For instance, after dressing with the antibiotic mixture to resist inflammatory reaction, or if the root apex of the upper posterior tooth is adjacent to maxillary sinus, it may be more difficult to induce enough blood.

Clinical regenerative endodontics has been focused on immature teeth because they have a greater chance of pulp tissue regeneration. It is believed that immature teeth with open apices allow more stem cells to migrate into root canals, and more stem cells found near immature root apices have been shown to possess a great regeneration potential in pulp regeneration [12]. Moreover, there are narrower apical pathways for cell migration in mature teeth, and sufficient disinfection is more challenging than that in immature teeth.

In conclusion, the present case demonstrates the resolution of clinical signs and symptoms with cementum-like tissue formed in the root canal and pulp vitality nearly recovered in the mature permanent premolar with pulpitis after regenerative endodontic treatment. Current regenerative procedures successfully produce root development, but still no evidence is there to reestablish a real pulp tissue. Higher level of evidence with large-scale investigations and randomized controlled clinical trials is still needed. Anyway, it might be possible that regenerative endodontic treatment could be widely used on mature teeth in the future and become an alternative to conventional endodontic therapies.

## Figures and Tables

**Table 1:** Recovery of pulp vitality at different follow-up time points.

Follow-up time points	Response to pulp testing
3 months	Not responsive
6 months	Nearly normal
12 months	Normal
30 months	Normal
